# Serial Lipocalin 2 and Oncostatin M levels reflect inflammation status and treatment response in axial spondyloarthritis

**DOI:** 10.1186/s13075-021-02521-y

**Published:** 2021-05-14

**Authors:** Florence W. L. Tsui, Aifeng Lin, Ismail Sari, Zhenbo Zhang, Hing Wo Tsui, Robert D. Inman

**Affiliations:** 1 Department of Immunology, University of Toronto, Toronto, Ontario Canada; 2 KeyIntel Medical Inc, Toronto, Ontario Canada; 3 Krembil Research Institute, University Health Network, Toronto, Ontario Canada; 4 Schroeder Arthritis Institute, University Health Network, Toronto, Ontario Canada; 5 Department of Internal Medicine, Faculty of Medicine, Dokuz Eylul University, Izmir, Turkey; 6 Department of Biomedical and Molecular Science, Queen’s University, Kingston, Ontario Canada; 7 Department of Medicine and Institute of Medical Sciences, University of Toronto, Toronto, Ontario Canada

**Keywords:** Axial spondyloarthritis, Lipocalin 2, Oncostatin M, MRI, Joint inflammation, Treatment response

## Abstract

**Background:**

Informative serum biomarkers for monitoring inflammatory activity and treatment responses in axial spondyloarthritis (axSpA) are lacking. We assessed whether Lipocalin 2 (LCN2) and Oncostatin M (OSM), both having roles in inflammation and bone remodeling, may accurately reflect chronic joint inflammation and treatment response in axSpA. Previous reports in animal models showed involvement of LCN2 and OSM in joint/gut inflammation. We asked whether they also play a role in human axSpA.

**Methods:**

We analyzed a longitudinal observational axSpA cohort (286 patients) with yearly clinical assessments and concurrent measurements of serum LCN2 and OSM (1204 serum samples) for a mean of 4 years. Biomarker levels were correlated with MRI scoring and treatment response.

**Results:**

Persistent and transient elevation of LCN2 and OSM were observed in axSpA patients. Persistent elevation of LCN2 or OSM, but not CRP, correlated with sacroiliac joint (SIJ) MRI SPARCC scores (Pearson’s correlation *p* = 0.0005 and 0.005 for LCN2 and OSM respectively), suggesting that LCN2/OSM outperforms CRP as reflective of SIJ inflammation. We observed both concordant and discordant patterns of LCN2 and OSM in relationship to back pain, the cardinal clinical symptom in axSpA. Twenty-six percent (73/286) of the patients remained both clinically and serologically active (CASA). Sixty percent (173/286) of the patients became clinically quiescent, with back pain resolved, but 53% (92/173) of them were serologically active (CQSA), indicating that pain control may not indicate control of joint inflammation, as reflected by positive MRI imaging of SIJ. With respect to treatment responses, transient elevation of LCN2 or OSM over time was predictive of better response to all treatments.

**Conclusion:**

In axSpA, persistent LCN2 and/or OSM elevation reflects chronic SIJ inflammation and suboptimal treatment response. In our cohort, half of the currently deemed clinically quiescent patients with back pain resolved continued to demonstrate chronic joint inflammation. LCN2 and OSM profiling outperforms CRP as a predictive measure and provides an objective assessment of chronic local inflammation in axSpA patients.

**Supplementary Information:**

The online version contains supplementary material available at 10.1186/s13075-021-02521-y.

## Background

Serological biomarkers which accurately reflect local joint inflammation and treatment response are lacking in axial spondyloarthritis (axSpA), a progressively debilitating disease. Both genetic and environmental effects such as microbial factors contribute to the pathogenesis of axSpA. We explored the role of Lipocalin 2 (LCN2) and Oncostatin M (OSM) in local inflammation of axSpA patients for the following reasons: First, LCN2 is an acute-phase protein released in response to microbial triggers [1] and has both pro- and anti-inflammatory properties which are context-dependent [2–5]. Elevated LCN2 levels have been reported in patients with IBD [6] and psoriasis [7], which are common axSpA comorbidities. We have recently shown that in patients with concurrent radiographic axSpA and IBD, elevated LCN2 was associated with coexisting ankylosis and gut inflammation [8]. Second, OSM is also an acute-phase protein [9]. In IBD, the primary association of TNFi non-responsiveness is with elevated levels of mucosal OSM [10]. Subclinical and clinical gut inflammation is common in axSpA (about 60% and 10% respectively). Since both LCN2 and OSM have known functions in local inflammation and bone remodeling [2, 5, 11, 12], the pathological hallmark of axSpA, we propose that both LCN2- and OSM-associated pathways are involved in axSpA pathogenesis. There is a mechanistic basis to support this perspective: *ank/ank* mice show higher serum LCN2 in mutant mice with gut involvement [8] and gut inflammation in mice is driven by OSM [10]. To address whether these inflammation mechanisms play a similar role in human axSpA pathogenesis, we conducted an observational study.

Traditionally, single point measurement of all relevant clinical parameters from large cohorts of axSpA patients has been used to decipher patterns with sophisticated statistical methods, and multiple clinical parameters have been used to reflect disease activity. The major limitations of this approach relate to the lack of understanding of the biological connectivity among the multiple factors identified and the inability to stratify patients into more homogeneous subgroups which is necessary for targeted therapies. An alternate approach has recently been used to implicate a possible role for endothelin-1 in systemic sclerosis using repeated measurements of serological markers [13]. We chose to use the latter approach and performed a longitudinal association study of 286 axSpA patients (200 radiographic axSpA and 86 non-radiographic-axSpA). We evaluated the association of LCN2 and OSM levels using the cardinal clinical symptom (back pain) in defining treatment response of these patients. By understanding the relationship of LCN2/OSM changes in response to treatment during the disease course, we established the range of LCN2/OSM levels that could be used to predict treatment response with single point baseline measurements. By identifying patients based on pathway involvement, patients with persistent OSM positivity need special attention in disease management, as current therapies are suboptimal to control joint inflammation in these patients.

## Methods

### Patients

Two hundred eighty-six axSpA patients (200 radiographic and 86 non-radiographic axSpA) followed yearly (as per protocol) for up to 12 years with serum banking and concurrent clinical parameters in a longitudinal observation axSpA were assessed. Suppl. Table 1 summarizes the demographic features of this cohort.

### Study approval

The study was approved by University Health Network (UHN) research ethics committee. All participating patients provided written informed consent. A written informed consent was received from participants prior to inclusion in the study. Participants were identified by study number in the analyses.

### Selection of homogeneous patient groups based on LCN2 or OSM profiles

Annual serial measurements of LCN2 and OSM levels (over a course of at least 4 years; 1204 samples, i.e., average ~4 serial samples per patient) revealed four groups of patients (Suppl. Table 2): (1) LCN2-elevated group with undetectable OSM levels, comprising 43% (123/286) of the cohort. The cut-off for LCN2 is 150ng/ml. (2) OSM-positive group with detectable OSM but normal LCN2 levels, comprising 9% (27/286) of the cohort; (3) LCN2-elevated group with detectable OSM levels, comprising 26% (74/286) of the cohort; and (4) LCN2 normal and OSM-negative group with no elevation in either biomarker, comprising 22% (62/286) of the cohort. In both L-elevated and O-positive groups, persistent and transient profiles are observed. Persistent elevation is defined as elevation of LCN2 (L++) or OSM (O++) levels which are sustained over a period of at least 2 years. Transient elevation is defined as a single elevation over a period ≥2 years (L+ and O+).

### MRI scoring

Among patients who had MRI assessments, 37 of them were used for association analysis. Spondyloarthritis Research Consortium of Canada (SPARCC) scoring and Berlin spinal joint scoring [14–16] were evaluated. Scoring was done independently by two readers (IS and SL). The mean scores were used for correlation analysis with LCN2 or OSM levels (Pearson’s correlation and Spearman’s rho [nonparametric] correlation). MRI taken within 12 months of the time of biomarker measurements were used for this analysis.

### Quantification of serum LCN2 and OSM levels by ELISA

The sequential samples (stored at −70°C) from each patient were thawed and analyzed at the same time to minimize assessment variabilities. Both LCN2 and OSM levels were measured by ELISA according to the manufacturer’s protocol (LCN2 ELISA kit: R & D Systems, DLCN20; OSM ELISA kit: Thermo Scientific, EHOSM). The mean minimum detectable limit for human LCN2 was 0.012ng/ml (R & D Systems), and the lower limit of detection for human OSM was 1pg/ml (Thermo Scientific). Previously, we used 100ng/ml as the cut-off for LCN2 (as determined by mean + 2SD of healthy controls) [8]. In this report, we used 150ng/ml to increase the specificity and positive predictive value (Suppl. Table 3). There are challenges in determining the cut-offs for OSM [17]. We use undetectable vs detectable OSM levels in this report.

### Statistics

One-way analysis of variance (ANOVA) and Student’s *t*-test were carried using the GraphPad Prism program. A *p*-value of less than 0.05 was considered significant. Data are presented as mean ± standard error. Calculators from socsistatistics.com were used for chi-square tests, Pearson’s correlation coefficient, and Spearman’s rho correlation.

## Results

### Elevated serum LCN2 and/or detectable OSM levels in axSpA patients

We previously reported that serum levels of LCN2 are elevated in radiographic axSpA patients [8]. In this report, we focus on axSpA patients with single pathway involvement, i.e., either with elevated LCN2 and no detectable OSM levels or with detectable OSM but normal LCN2 levels. Since we have sequential samples for each patient, the highest level from each patient (usually from the first visit) was used for comparisons among subgroups.

#### Patients with elevated LCN2 but no detectable OSM levels

**Fig. 1:**
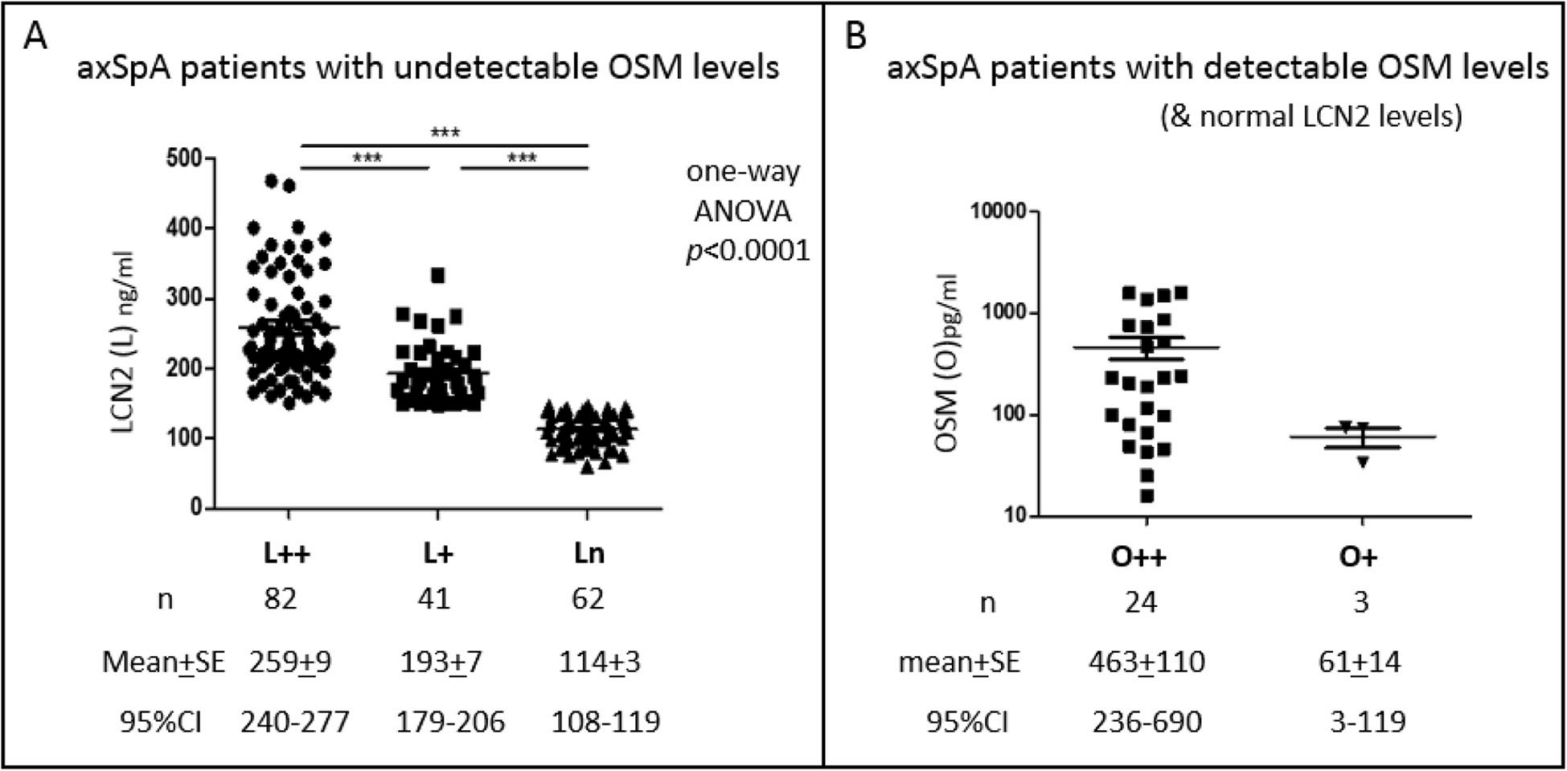
LCN2 or OSM levels in axSpA patients with single pathway involvement. **a** axSpA patients with undetectable OSM levels: LCN2 levels in patients with persistent (L++) vs transient (L+) vs normal LCN2 (Ln). **b** axSpA patients with detectable OSM but normal LCN2 levels: OSM levels in patients with persistent (O++) vs transient OSM (O+). The highest LCN2/OSM levels from the serial measurements of each patient were used for comparisons in the plots. One-way analysis of variance (ANOVA) followed by Bonferroni’s multiple comparison and Student’s *t* test were used to determine significance

Significantly higher LCN2 levels (mean ± SE) are found in those with persistent LCN2 (L++) vs transient (L+) elevation (L++ vs L+ 259 ng/ml ± 9 vs 193 ng/ml ± 7; *p* < 0.0001). Ln patients have normal LCN2 levels (114 ng/ml ± 3; 150 ng/ml being the cut-off) and undetectable OSM levels (Fig. 1a). Importantly, there are no overlaps in the 95% confidence intervals (CIs) among the 3 subgroups (240–277ng/ml for L++, 179–206ng/ml for L+, and 108–119ng/ml for Ln). These ranges could be helpful for the assessment of single-point baseline levels to predict the likelihood of whether LCN2 levels could be persistent as the disease proceeds.

#### Patients with detectable OSM but normal LCN2 levels

Higher OSM levels are found in those with persistent OSM (O++) vs transient (O+) elevation (O++ vs O+ 463 pg/ml ± 110 vs 61 pg/ml ± 14), though it is not significantly different. This may be due to the spread of levels in O++ patients and too few O+ patients for the comparison (Fig. 1b). There are also no overlaps in the 95% CI between the 2 subgroups (236–690pg/ml for O++; 3–119pg/ml for O+). Based on the non-overlapping CI levels, baseline levels might reflect whether the detectable levels could persist as disease proceeds.

### MRI evidence of sacroiliac joint (SIJ) involvement is correlated with elevated LCN2 and OSM in axSpA patients

**Fig. 2:**
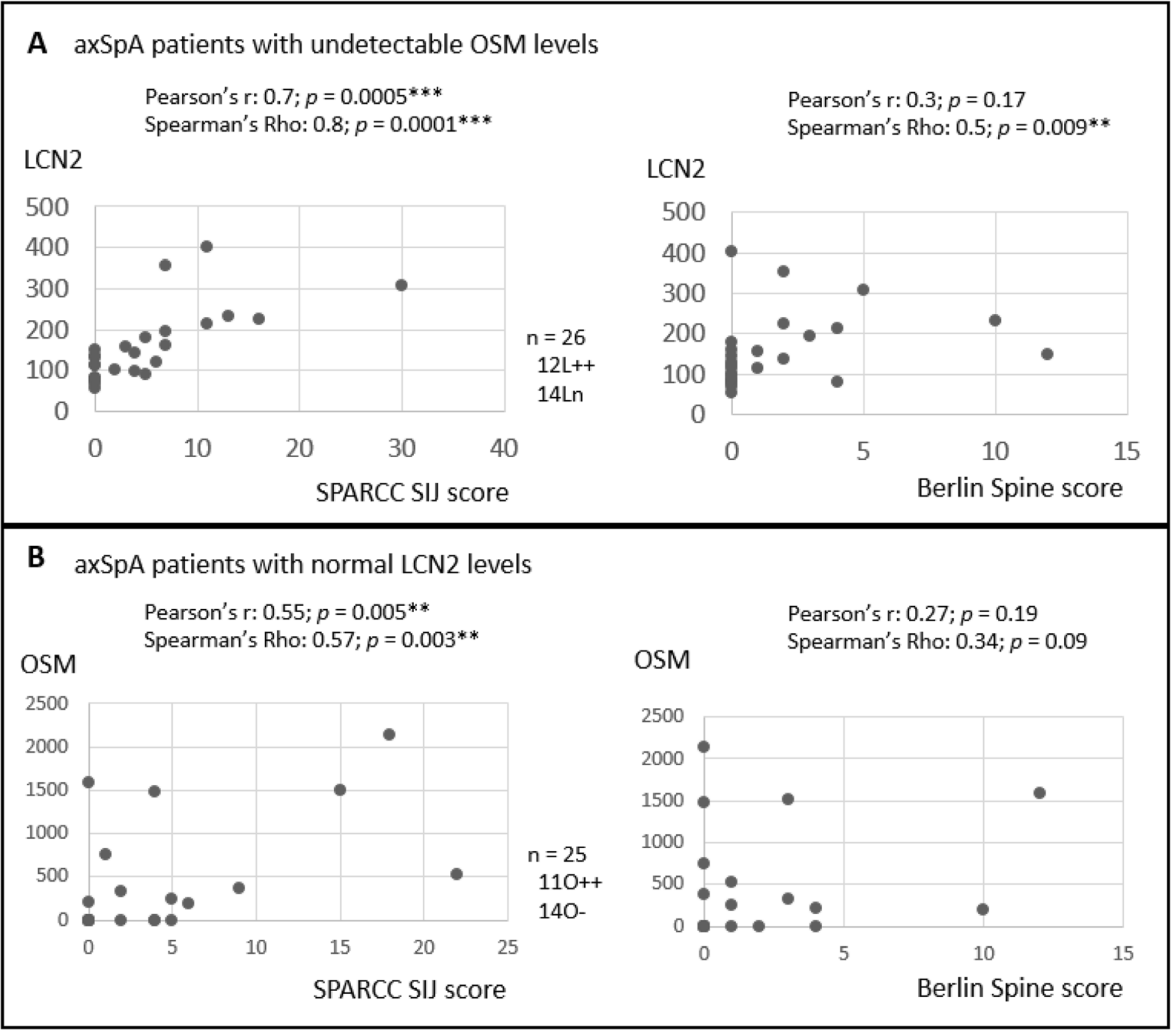
Correlation of LCN2 or OSM levels with MRI scores. **a** Correlation of LCN2 levels with SPARCC SIJ scores and Berlin spine scores in patients with persistently elevated LCN2 (L++) and those with normal LCN2 (Ln). OSM is undetectable in these patients. **b** Correlation of OSM levels with SPARCC SIJ scores and Berlin spine scores in patients with persistent OSM elevation (O++) and those with undetectable OSM (O−). LCN2 is normal in these patients. Pearson’s correlation coefficient test and Spearman’s rho correlation calculation were used to determine significance

We previously showed that there is a relationship between circulating LCN2 and spinal ankylosis in radiographic axSpA patients [8]. In this study, we asked whether LCN2/OSM plays a role in SIJ and/or spinal inflammation. For this purpose, we selected axSpA patients with no spinal ankylosis (mSASS=0). As both LCN2 and OSM can contribute to joint inflammation, to define their roles in inflammation separately, we focus our comparison among 3 patient subgroups: those with persistent LCN2 elevation (L++; *n*=12) vs those with persistent OSM positivity (O++; *n*=11) vs those with normal LCN2 and undetectable OSM (LnO−; *n*=14). For L++ and Ln patients, significant correlations are present between LCN2 levels and SPARCC SIJ scores (Fig. 2a; Pearson’s correlation coefficient = 0.7, *p* = 0.0005; Spearman’s rho = 0.8, *p* = 0.0001). Similarly, for O++ and O− patients, there is a significant correlation between OSM levels and SPARCC SIJ scores (Pearson’s correlation coefficient = 0.55, *p* = 0.005; Spearman’s rho 0.57, *p* = 0.003; Fig. 2b). Using Pearson’s correlation analysis, there is no correlation between LCN2 or OSM levels and Berlin spine scores in the respective patient groups. However, for L++ and Ln patients, there is a significant correlation between LCN2 levels and Berlin spine scores using Spearman’s rho correlation analysis (Spearman’s rho 0.5, *p* = 0.009). These results suggest that persistent elevation of LCN2 (L++) and persistent elevation of OSM (O++) are associated mainly with SIJ inflammation.

There is no correlation between MRI SPARCC SIJ scores and CRP levels (Suppl. Figure 1). In this regard, LCN2 and OSM outperform CRP.

### Association of treatment outcome with pathway involvement

Since persistent elevation of LCN2 (L++) or OSM (O++) reflects SIJ inflammation (Fig. 2), we asked whether there is any association of LCN2 or OSM levels with the central clinical symptom back pain in the evaluation of treatment response of the patients. Using sequential LCN2 and OSM measurements and sequential back pain scores (question 2 of the BASDAI) [18], concordant vs discordant association was observed in responses to all treatments (both with or without TNFi; patients who never received TNFI were treated with NSAIDs/DMARDs). Patients with the concordant pattern include those who were clinically quiescent and serologically quiescent (**CQSQ**) vs clinically active (back pain score >4) and serologically active (**CASA**). **CQSQ** patients had normal LCN2 or undetectable OSM levels as well as reduced back pain scores (<4) after treatments. **CASA** patients remained having persistently elevated LCN2 and/or OSM and back pain scores (>4) after treatments. Patients with the discordant pattern include those who were **CQSA** vs **CASQ**. **CQSA** patients had back pain resolved but remained serologically active. **CASQ** patients had persistent back pain even though they were serologically quiescent. Persistent back pain in **CASQ** patients is likely not due to involvement of LCN2 and OSM pathways. Alternatively, it could reflect a possibly non-inflammatory nature of back pain in **CASQ** patients.

We questioned how the pathways involving LCN2 or OSM might affect the outcome of treatments. We first compared treatment response in patients having involvement of the LCN2 pathway alone (no detectable OSM present). For patients with persistent LCN2 elevation (L++), profiling indicated that both concordant and discordant patterns were observed. Patients with concordant response were predominantly **CASA** (35% [29/82]). Only 12% [10/82] are **CQSQ**. Most of the patients with discordant response are **CQSA** (pain resolved but LCN2 remained elevated; 50% [41/82]). Only two patients with discordant response are **CASQ** (pain persisted but with normal LCN2; 2% [2/82]; Table 1A left panel). Similarly, both concordant and discordant treatment responses are observed in patients with transient LCN2 elevation (L+). However, for patients with concordant treatment responses, significantly more of them are responders [**CQSQ**] (L++ vs L+; **CQSQ** 12% vs 71%; **CASA** 35% vs 10%; *χ*^2^ = 27.9, *p*<0.00001; Table 1A right panel).

**Table 1:**
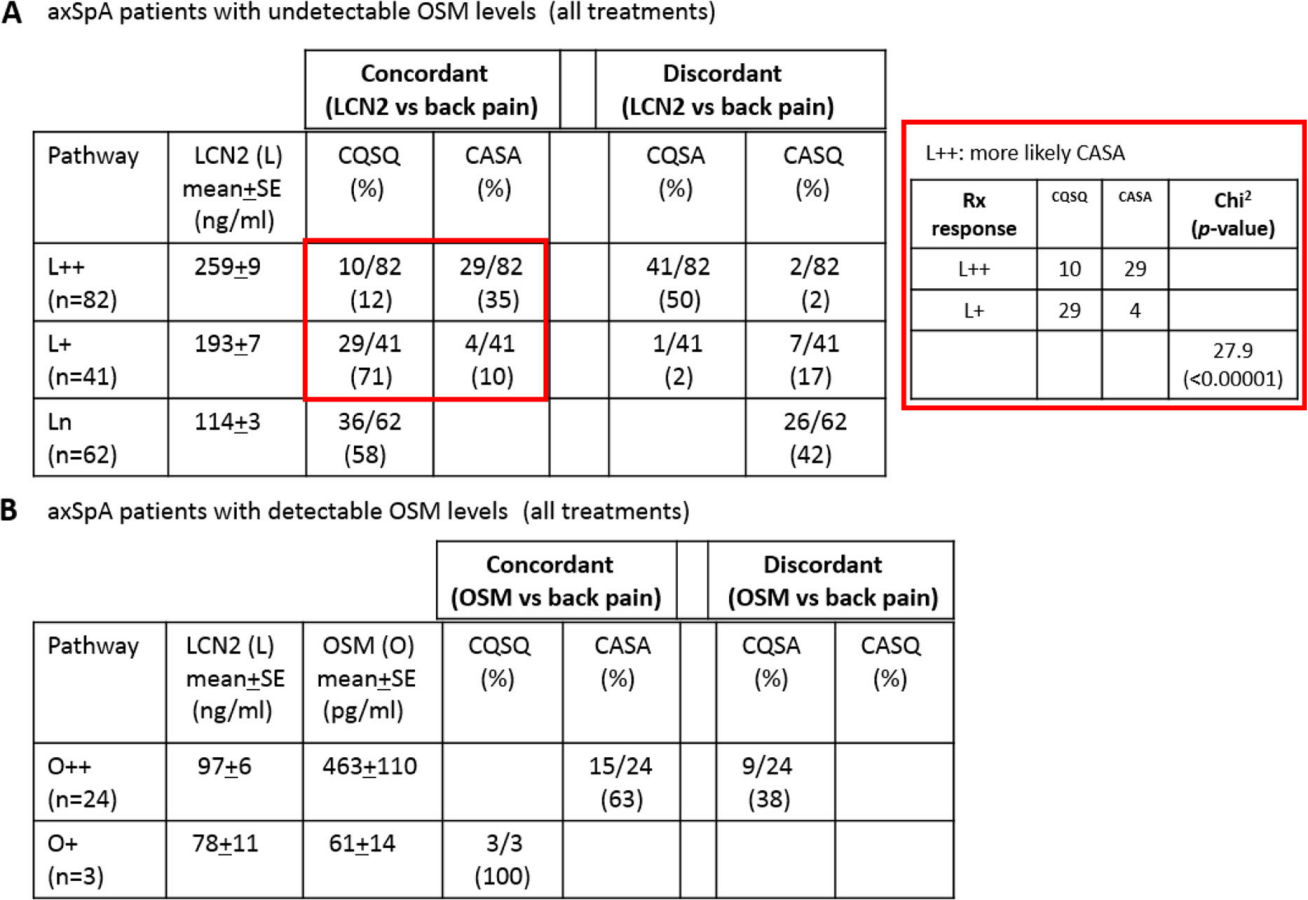
Association of treatment outcome with LCN2 and OSM in axSpA with a single pathway involvement. (A) *Left panel*: Comparison of treatment outcome in patients with persistent LCN2 elevation (L++) vs transient LCN2 elevation (L+) vs normal LCN2 (Ln). OSM was undetectable in these patients. *Right panel*: Comparison of treatment outcome in patients with persistent LCN2 elevation (L++) vs transient LCN2 elevation (L+). Pearson’s chi-square test was used to determine significance. (B) Comparison of treatment outcome in patients with persistent OSM positivity (O++) vs transient OSM positivity (O+). CQSQ, clinically quiescent and serologically quiescent; CASA, clinically active and serologically active; CQSA, clinically quiescent and serologically active; CASQ, clinically active and serologically quiescent

Out of 62 LnO− patients, 58% (36/62) are deemed responders [**CQSQ**] as defined by normal LCN2, undetectable OSM, and low back pain scores. The remaining 42% (26/62) showed discordant treatment response, having the **CASQ** pattern as both LCN2 and OSM were persistently normal, but back pain persisted (Table 1A left panel).

Profiling treatment responses in patients with involvement of OSM (O) pathway alone (O++ and O+) revealed differences between the two subgroups. All O++ patients with concordant treatment responses are **CASA** (63% [15/24]; with persisting elevated OSM levels and back pain >4). Those with discordant treatment response are all **CQSA** (pain resolved but elevated OSM levels persisted; 38% [9/24]; Table 1B). Unlike O++ patients, all O+ patients in our cohort (*n*=3) are **CQSQ** with both pain resolved and OSM levels normalized. Thus, among patients with involvement of only one pathway (either LCN2 or OSM alone; comprising 43% and 9% of our cohort respectively; suppl. Table 2), transient elevation of LCN2 or OSM during the disease course appears to be an indicator of better response to all treatments (Table 1A and B). In addition, current treatments are less effective in O++ patients. None of the 24 O++ patients in our cohorts was **CQSQ**, although about half of them are **CQSA** with back pain resolved but OSM elevation persisted (Table 1B).

Results on treatment response based on patients receiving TNFi vs no TNFi (but with NSAIDS and/or DMARDs) are summarized in Suppl. Tables 4 and 5. In general, treatment response profiles (with vs without TNFi) are similar. However, for TNFi-treated patients with LCN2 involvement only, L++ patients with concordant treatment response, compared to L+ patients, significantly more L+ patients are responders [**CQSQ**] (L++ vs L+; **CQSQ** 12% vs 74%; **CASA** 44% vs 6%; *χ*^2^ = 28.5, *p*<0.00001; Suppl. Table 4A). Though the trend is similar in patients not treated with TNFi, there are no significant differences. This implicates TNFi as being more effective in patients with LCN2 pathway involvement.

Profiling of response treatments in radiographic vs non-radiographic axSpA patients showed similar patterns (data not shown). We also addressed whether HLA B27 status, gender, and comorbidities affected the outcome of treatment response by biomarker profiling and found that none of these cofactors affected the analyses.

### MRI evidence of joint inflammation in CQSA patients

**Fig. 3:**
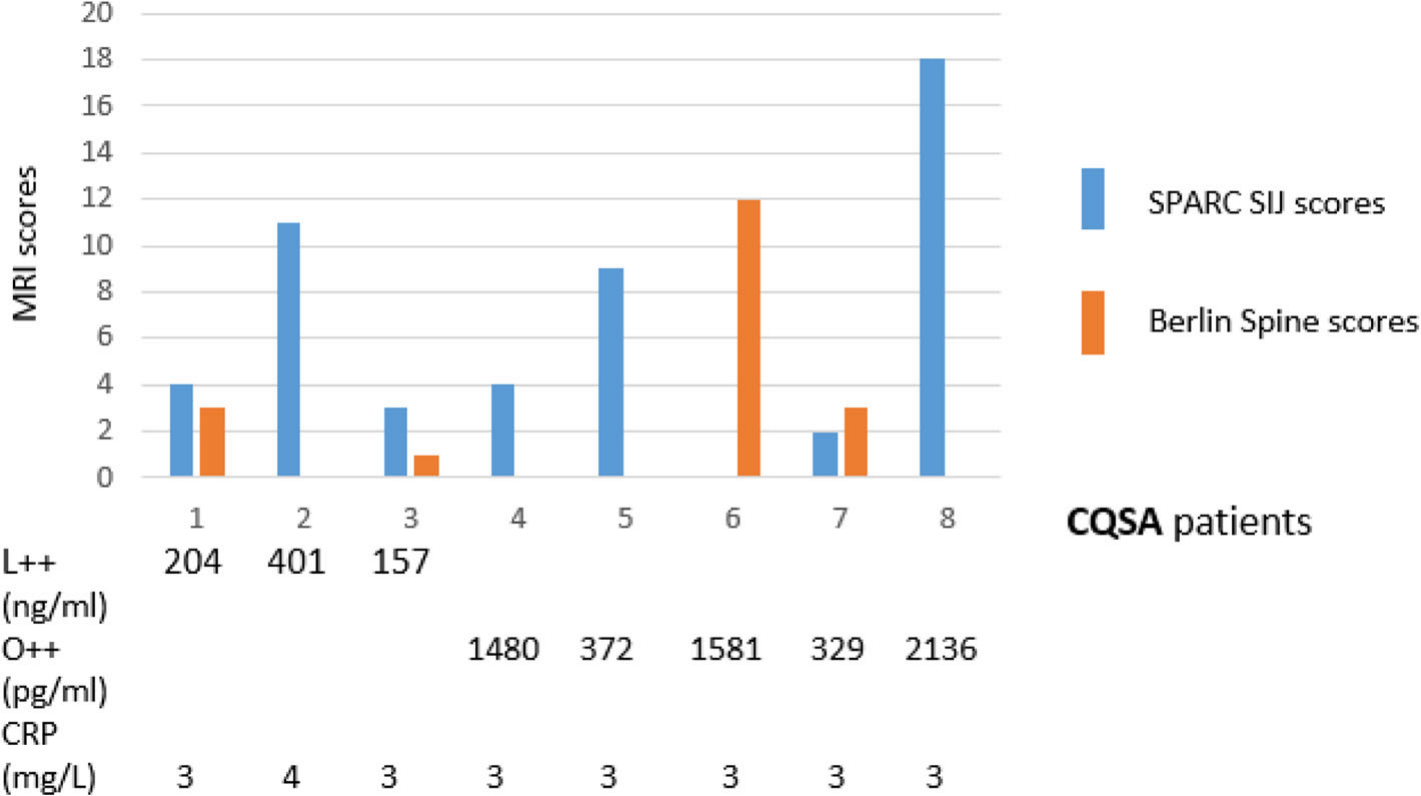
MRI inflammatory scores in CQSA patients. SPARCC SIJ scores (blue bars) and Berlin spine scores (orange bars) are charted for each of the 8 **CQSA** patients. The levels of LCN2/OSM/CRP measured at the time of MRI assessment were noted below the chart. Three of them had persistently elevated LCN2 (L++) and 5 had persistently elevated OSM (O++). None of them had elevated CRP in the same time point measurement

Patients who were deemed clinical quiescent and serologically active (**CQSA**) are prominently L++ and O++ (Table 1) even though back pain has resolved. We asked whether these patients had joint inflammation as revealed by MRI inflammatory scores. Of those **CQSA** patients with concurrent MRI scores and LCN2/OSM levels, 88% (7/8) had positive MRI SIJ SPARCC scores, suggesting that persistently elevated LCN2 (L++) or persistently elevated OSM (O++) levels are associated with SIJ inflammation (Fig. 3). The single **CQSA** patient who had negative MRI SIJ SPARCC scores had positive Berlin spine scores. None of these patients had elevated CRP in the same time point measurement.

### A novel perspective related to defects leading to axSpA development

**Fig. 4:**
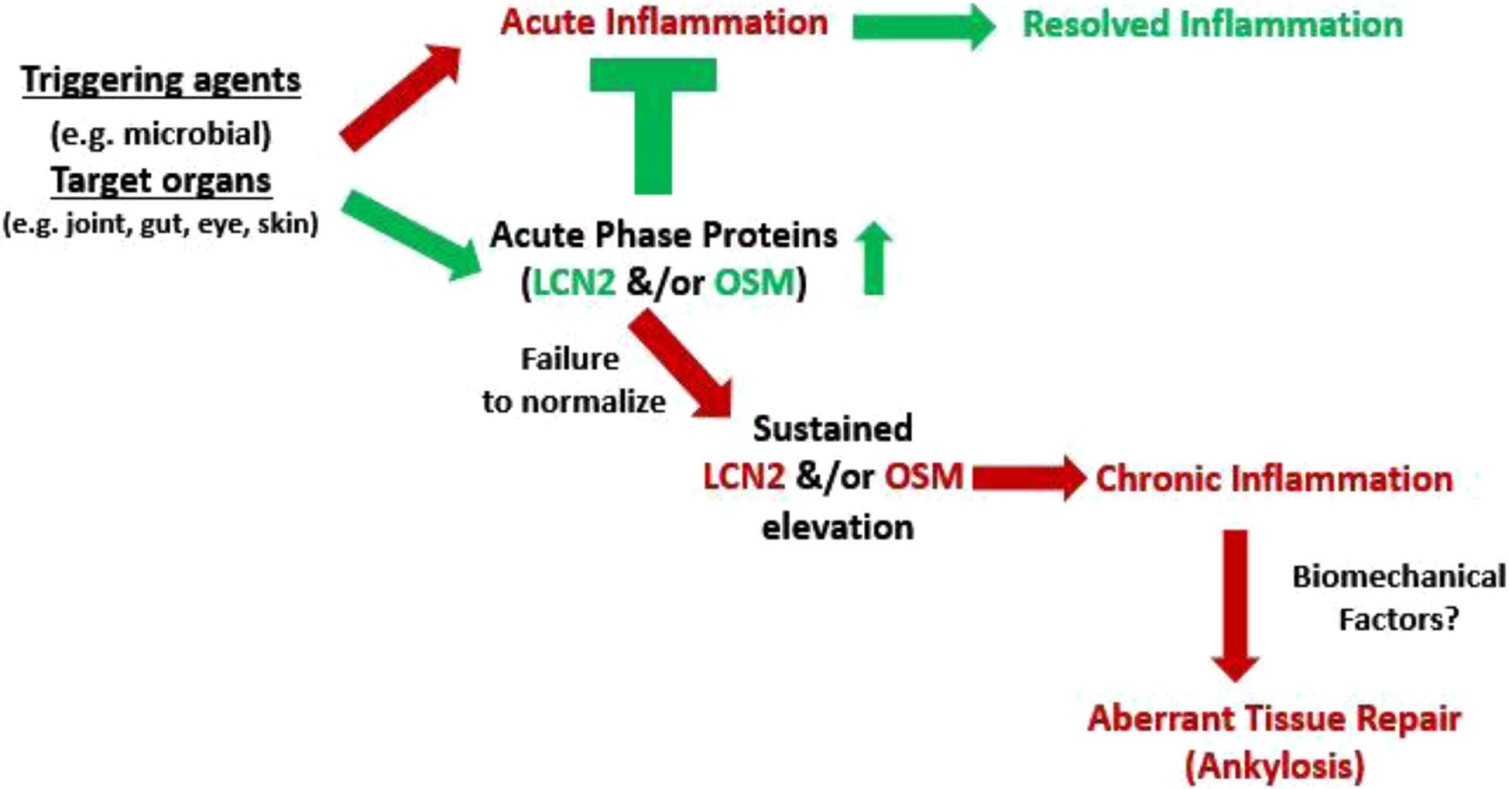
A novel perspective for axSpA development. Upon external triggering agents, acute-phase proteins (including LCN2 and OSM) are produced by the innate immune system to combat acute infection. Transient elevation of these proteins is thought to play a protective role in host defense. Nevertheless, failure to normalize these self-defense proteins in a timely manner would cause local chronic inflammation. Eventually, it leads to excessive tissue repair, including ankylosis in spondylitis

Acute-phase proteins (APPs) play a prominent role in the defense mechanisms of the host innate immune system. Both LCN2 and OSM are known APP [9, 19, 20]. Transient elevation of APP is thought to play a protective role in host defense. However, failure to normalize APP in a timely manner would have pro-inflammatory consequences, resulting in chronic inflammation. An important implication of this hypothesis is that persistently elevated APP such as LCN2 and OSM leads to SIJ inflammation as reflected by the MRI results. Figure 4 shows a schematic of this perspective.

CRP, an APP, contributes to systemic inflammation [21]. To further evaluate our hypothesis, we asked whether persistent elevation of CRP could be found in radiographic axSpA (or ankylosing spondylitis [AS]) patients in our cohort. Nr-axSpA patients are excluded in this analysis as only 2 patients had elevated CRP in our cohort.

**Fig. 5:**
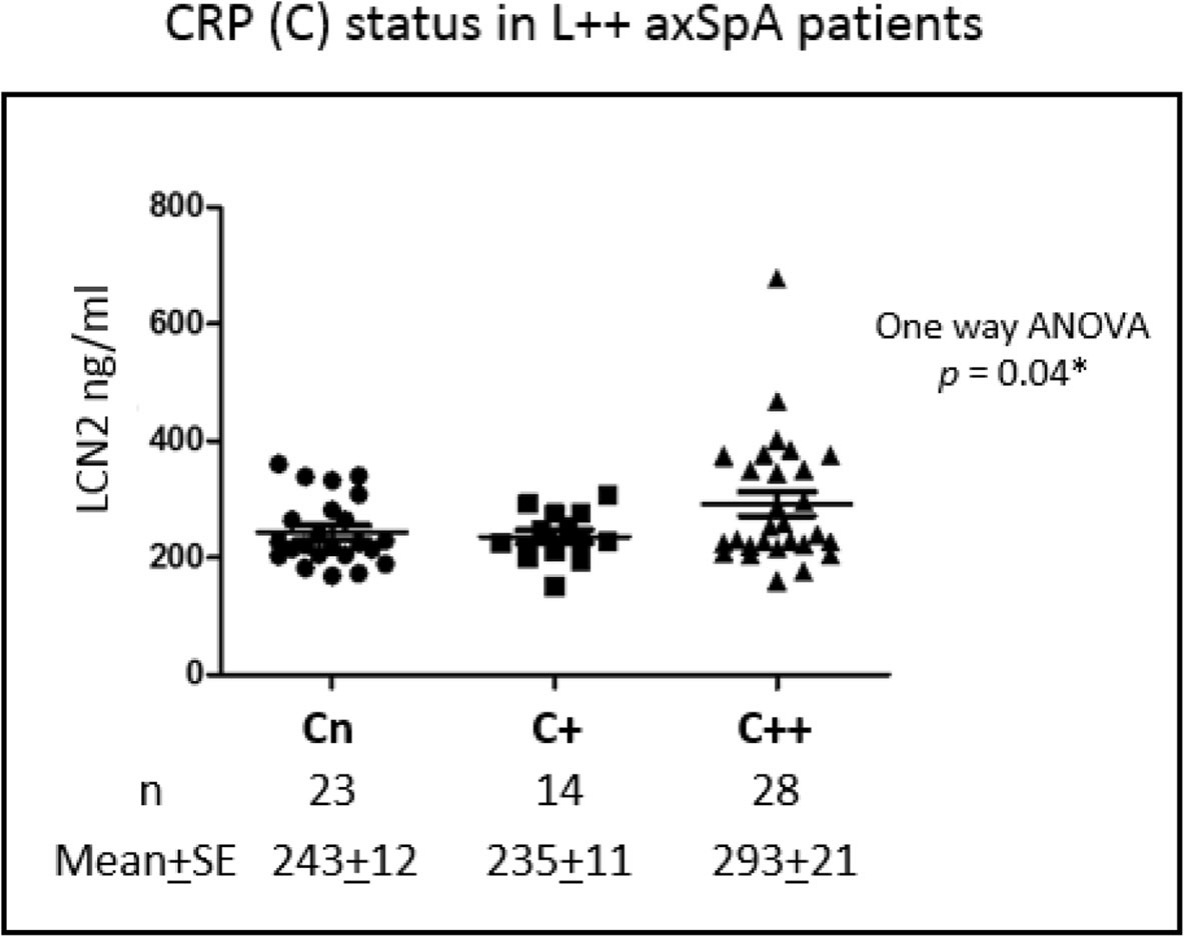
Detection of LCN2 and CRP levels in AS patients with persistently elevated LCN2 (L++). LCN2 levels were compared in patients with normal CRP (Cn) vs transient CRP elevation (C+) vs persistent CRP elevation (C++). One-way analysis of variance (ANOVA) followed by Bonferroni’s multiple comparison test and Student’s *t* test were used to determine significance

Similar to LCN2 and OSM, both transient and persistent elevations of serum CRP are found in AS patients as expected. Patients with any CRP elevation (C++ or C+) are predominantly found in patients with persistent LCN2 elevation (L++). Among these patients, 43% [28/65] and 22% [14/65] were C++ and C+ respectively. C++ patients had higher LCN2 levels (compared to levels from C+ and Cn patients; 293 ng/ml ± 21 [C++] vs 235ng/ml ± 11 [C+] vs 243 ng/ml ± 12 [Cn]; one-way ANOVA *p* = 0.04; Fig. 5).

We asked whether persistent vs transient CRP elevation might have differential effects on treatment response (all treatments) in L++ patients (**C++** vs **C+** vs Cn). **C++** patients have higher and lower percentage of **CASA** and **CQSQ** patients respectively (**CASA** 36% vs 21%; **CQSQ** 7% vs 29%; C++ vs C+ respectively; Table 2A); though there is no significant difference (Table 2B). Taken together, persistent CRP elevations (C++) likely have a small effect, if any, on treatment response outcome in L++ patients with no detectable OSM.

**Table 2:**
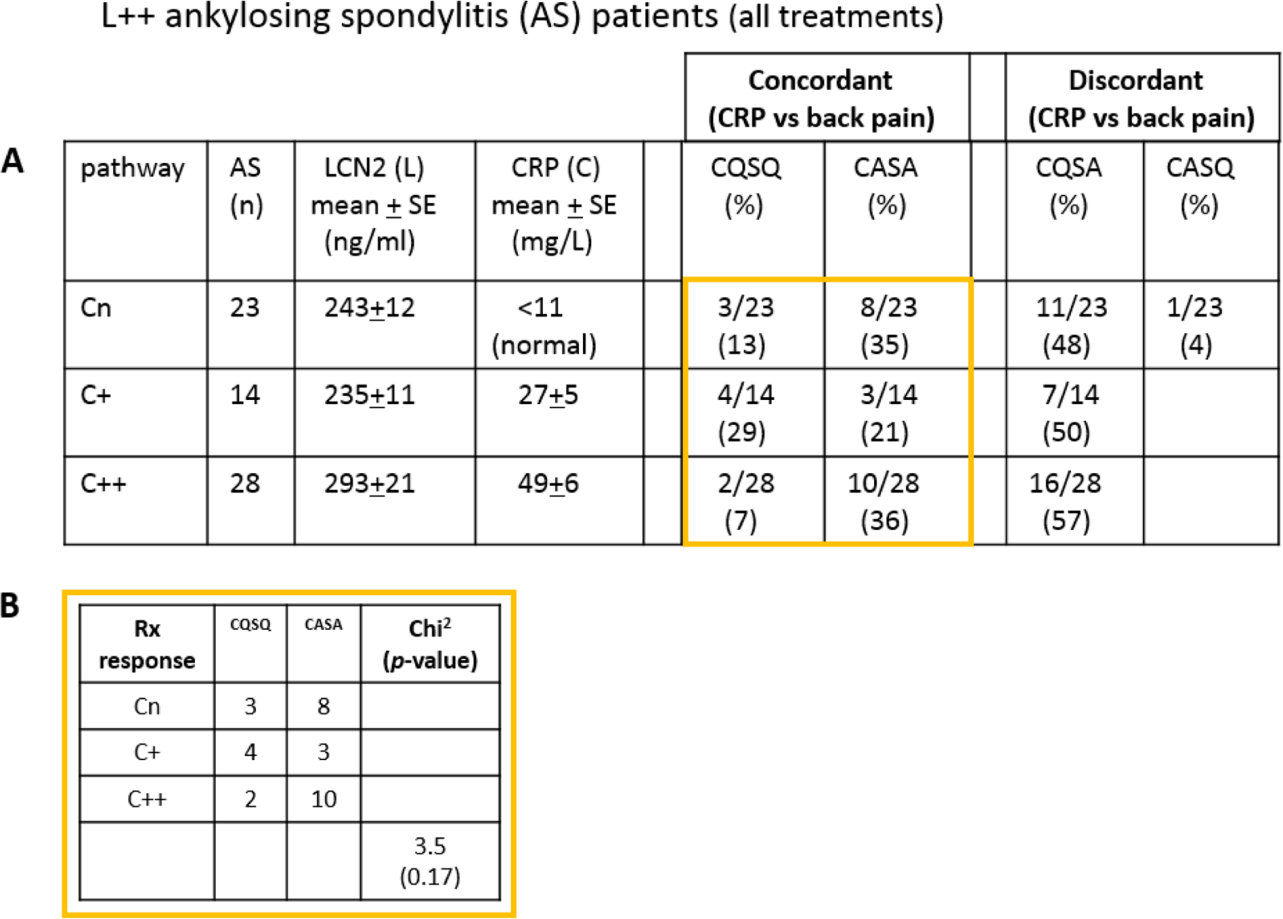
Association of treatment outcome with CRP in AS patients with persistent LCN2 elevation (L++). (A) Comparison of treatment outcome in patients with normal CRP (Cn) vs transient CRP elevation (C+) vs persistent CRP elevation (C++). (B) Pearson’s chi-square test was used to determine significance. CQSQ, clinically quiescent and serologically quiescent; CASA, clinically active and serologically active; CQSA, clinically quiescent and serologically active; CASQ, clinically active and serologically quiescent

## Discussion

This report has important findings which have an impact on improving axSpA disease management: Firstly, the identification of involvement of two APPs, LCN2 and OSM, acting singly or in combination, in axSpA. Elevated baseline LCN2 [8] and OSM [22] levels were reported in axSpA patients. Here, we showed two patterns of LCN2 or/and OSM levels. Persistent elevation of LCN2 alone (L++; 43%, 123/286) is more prevalent than persistent elevation of OSM alone (O++; 9%, 27/286). There are no overlaps in the 95% CI range between persistent vs transient L/O elevation. This could be used to predict whether the patient might have persistent L/O elevation based on the baseline level (Suppl. Table 6). Validation of this prediction is needed. In addition, it remains to be investigated, using animal studies, whether transient vs persistent elevation of LCN2 or OSM has opposing outcomes relating to inflammation (resolution of inflammation vs development of chronic inflammation).

Secondly, the first demonstration that LCN2 and OSM levels correlated with MRI SPARCC SIJ scores and thus reflect SIJ inflammation, the cardinal feature of axSpA. This report is focused on the relationship of LCN2 and OSM and chronic inflammation. This is the reason why we chose patients with no spinal ankylosis (mSASS=0) to analyze the correlation of LCN2 or OSM levels with MRI inflammation scores. As low numbers of patients with single pathway involvement and MRI assessment were available for this study (*n*=23), a larger study is warranted. Though MRI is currently the most sensitive tool for the detection of joint inflammation, it is not without limitations such as false positives for patients with low back pain. The use of LCN2 and OSM monitoring serves as a pre-screen to determine whether the costly MRI is needed to confirm findings from LCN2/OSM profiling.

CRP reflecting systemic inflammation has no correlation with MRI SPARCC SIJ scores (Suppl. Fig. 1) and thus is not a biomarker for local joint inflammation. CRP elevation is infrequently found in nr-axSpA patients [23], but is elevated in less than 30% of radiographic axSpA/ankylosing spondylitis (AS) patients with active disease [24]. Importantly, LCN2/OSM assessments outperform CRP and provide a convenient and objective assessment of chronic local inflammation in not only radiographic but also nr-axSpA patients.

Thirdly, the demonstration that profiling of LCN2 and OSM levels, together with concurrent back pain scores during the disease course, can effectively predict treatment responses in axSpA patients. Our clinical-serological approach has similarly been used in two diseases: SSc [25] and SLE [26, 27]. CQSA patients were identified in SLE. Our axSpA patients with discordant treatment responses (**CQSA**) had back pain resolved but LCN2 or OSM remained elevated. In our MRI inflammatory scores vs serological level correlations, 88% (7/8) **CQSA** patients had positive MRI SIJ SPARCC scores, and the single CQSA patient who had negative MRI SIJ SPARCC scores had positive Berlin spine scores, suggesting that persistent LCN2 or OSM levels are associated predominantly with SIJ inflammation (Fig. 2).

Fourthly, current axSpA management focuses on symptom (back pain) control. Of the 173 **CQ** patients in this study, 47% (81/173) were **SA** and 9 of them (11%) had mSASS >50. This is in contrast to only 3% (3/92) **CQSQ** patients who had mSASS>50 (*p* = 0.04; Suppl. Table 7). **CQSA** and **CASA** patients had a similar prevalence of patients with mSASS>50 (11% and 13% respectively), indicating that though back pain may be controlled (**CQ**), the disease may progress if LCN2/OSM continues to be elevated (**SA**). Thus, LCN2 and OSM profiling provides personalized and more effective treatment.

Finally, by grouping patients based on pathway involvement, patients with more homogeneous features can be identified. This facilitates more appropriate and targeted therapy. For example, we showed that current treatments are less effective in O++ patients. None of the 24 O++ patients in our cohorts was **CQSQ**, although about half of them are **CQSA** with back pain resolved but OSM elevation persisted (Table 1B). It remains to be evaluated whether anti-OSM could be an appropriate therapy for these O++ axSpA patients.

How would LCN2 and OSM mediate the link between the local gut and joint inflammation? Our hypothesis is that bacterial triggers in the gut/joint lead to persistent elevation of LCN2/OSM resulting in chronic local inflammation (Fig. 4). LCN2 and OSM can each act at multiple steps in gut and joint inflammation. Our results on MRI correlation with circulating LCN2 or OSM suggest that circulating LCN2/OSM reflects SIJ inflammation in axSpA patients. However, it has recently been shown that serum OSM did not correlate with mucosal OSM in IBD patients [17], suggesting that circulating OSM might be a better reflection of inflammation in the joint than in the gut in axSpA patients. Our current protocol does not include routine colonoscopy in our axSpA cohort. It remains to be investigated in future studies whether circulating LCN2 or OSM levels correlate with gut inflammation as detected by colonoscopy.

Published literature relating mainly to gut inflammation suggests that the LCN2 pathway is one of the targets for TNFi [6]. It also reflects the inability of TNFi alone to resolve LCN2-mediated inflammation. While TNFi block TNFα, other cytokines may persist to maintain the inflammatory process. IL17 synergizes with IL22 and TNFα to induce LCN2 expression in the colonic epithelium [6] as well as in bone cells [28]. To date, TNFi and IL17i agents seem comparable in clinical efficacy in axSpA [29]. Profiling LCN2 and OSM in axSpA patients would facilitate more systematic evaluations of medications which could be optimal for different patient subgroups.

There are limitations in retrospective studies, as the data are dependent on available information and our analyses were complicated by some gaps in clinical information. Back pain, a subjective assessment, is used as the key clinical decision-making. As the gold standard is challenging in axSpA, LCN2 and OSM monitoring can help in this respect. Prospective studies with well-designed parameters are needed to evaluate more rigorously the power and the limitations of LCN2 and OSM as early biomarkers for chronic local inflammation in axSpA patients.

## Conclusions

In axSpA, persistent LCN2 and/or OSM elevation reflects chronic SIJ inflammation and suboptimal treatment response. In our cohort, half of the currently deemed clinically quiescent patients with back pain resolved continued to demonstrate chronic inflammation. Importantly, LCN2 and OSM profiling outperforms CRP and provides a convenient and objective assessment of chronic local inflammation in axSpA patients. Together with concurrent back pain, LCN2 and OSM profiling provides precision management of axSpA.

## Supplementary Information

**Additional file 1: Figure S1.** Correlation of CRP levels with MRI scores. Correlation of CRP levels with SPARCC SIJ scores and Berlin Spine scores in patients with the involvement of LCN2 pathway alone (L++) and with normal LCN2 (Ln). Pearson's correlation coefficient test was used. **Table S1.** Demographics of axSpA patients in different categories. **Table S2.** Summary of different categories of patients with the involvement of different pathway(s). **Table S3.** Sensitivity, specificity, positive and negative predictive values using different LCN2 cutoffs. **Table S4.** Treatment outcome in patients with LCN2 pathway alone (TNFi vs no TNFi). A. Comparison of OSM negative patients treated with TNFi: patients with persistent LCN2 elevation (L++) vs. transient LCN2 elevation (L+) vs. normal LCN2 (Ln). B. Comparison of OSM negative patients never received TNFi treatment: L++ vs. L+ vs. Ln patients. One-way analysis of variance followed by Bonferroni’s multiple comparison test and Pearson’s chi square test were used. **Table S5.** Treatment outcome in patients with OSM pathway alone (TNFi vs no TNFi). A. Comparison of patients with normal LCN2 treated with TNFi: patients with persistent OSM elevation (O++) vs transient OSM elevation (O+). LCN2 and OSM levels were compared between these two patient groups. B. Comparison of patients with normal LCN2 and never received TNFi treatment: O++ vs O+ patients. LCN2 and OSM levels were compared between these two patient groups. **Table S6.** The use of 95%CI of LCN2 and OSM to predict signature of axSpA patients. **Table S7.** Prevalence of patients having mSASS>50 in **CQSA** vs **CQSQ** subgroups.

## Data Availability

Data sharing is not applicable to this article as no datasets were generated or analyzed during the current study.

## Abbreviations

APP: Acute-phase protein
ANOVA: One-way analysis of variance
AS: Ankylosing spondylitis
axSpA: Axial spondyloarthritis
BASDAI: Bath Ankylosing Spondylitis Disease Activity Index
CASA: Clinically active and serologically active
CASQ: Clinically active and serologically quiescent
CQSA: Clinically quiescent and serologically active
CQSQ: Clinically quiescent and serologically quiescent
CRP: C-reactive protein
C++: Persistent CRP elevation
C+: Transient CRP elevation
IBD: Inflammatory bowel disease
LCN2: Lipocalin 2
Ln: Normal LCN2 level
LnO−: Normal LCN2, negative OSM (undetectable) levels
L++: Persistent elevation of LCN2
L+: Transient elevation of LCN2
MRI: Magnetic resonance imaging
mSASSS: Modified Stoke AS Scoring System
nr-axSpA: Non-radiographic axial spondyloarthritis
OSM: Oncostatin M
O−: Negative OSM (undetectable) level
O++: Persistent elevation of OSM
O+: Transient elevation of OSM
SD: Standard deviation
SE: Standard error
SIJ: Sacroiliac joint
SPARCC: Spondyloarthritis Research Consortium of Canada
TNFi: Tumor necrosis factor inhibitor
UHN: University Health Network
95% CI: 95% confidence interval
