# Supplementary material for: Serial Lipocalin 2 and Oncostatin M levels reflect inflammation status and treatment response in axial spondyloarthritis
Source: Arthritis Res Ther. 2021 May 14;23:141. doi: 10.1186/s13075-021-02521-y (PMC8120829; doi:10.1186/s13075-021-02521-y)

**Additional file 1: Figure S1.** Correlation of CRP levels with MRI scores. Correlation of CRP levels with SPARCC SIJ scores and Berlin Spine scores in patients with the involvement of LCN2 pathway alone (L++) and with normal LCN2 (Ln). Pearson's correlation coefficient test was used. **Table S1.** Demographics of axSpA patients in different categories. **Table S2.** Summary of different categories of patients with the involvement of different pathway(s). **Table S3.**  Sensitivity, specificity, positive and negative predictive values using different LCN2 cutoffs. **Table S4.** Treatment outcome in patients with LCN2 pathway alone (TNFi vs no TNFi). A. Comparison of OSM negative patients treated with TNFi: patients with persistent LCN2 elevation (L++) vs. transient LCN2 elevation (L+) vs. normal LCN2 (Ln). B. Comparison of OSM negative patients never received TNFi treatment: L++ vs. L+ vs. Ln patients. One-way analysis of variance followed by Bonferroni’s multiple comparison test and Pearson’s chi square test were used. **Table S5.** Treatment outcome in patients with OSM pathway alone (TNFi vs no TNFi). A. Comparison of patients with normal LCN2 treated with TNFi: patients with persistent OSM elevation (O++) vs transient OSM elevation (O+). LCN2 and OSM levels were compared between these two patient groups. B. Comparison of patients with normal LCN2 and never received TNFi treatment: O++ vs O+ patients. LCN2 and OSM levels were compared between these two patient groups. **Table S6.** The use of 95%CI of LCN2 and OSM to predict signature of axSpA patients. **Table S7.** Prevalence of patients having mSASS>50 in **CQSA** vs **CQSQ** subgroups.

**Figure S1**

**
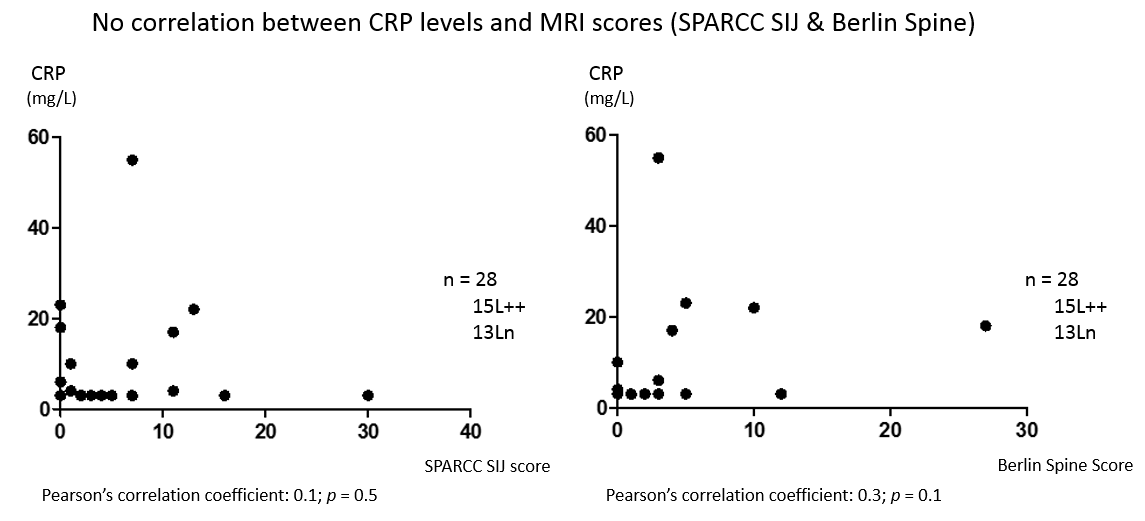
**

**Table S1**


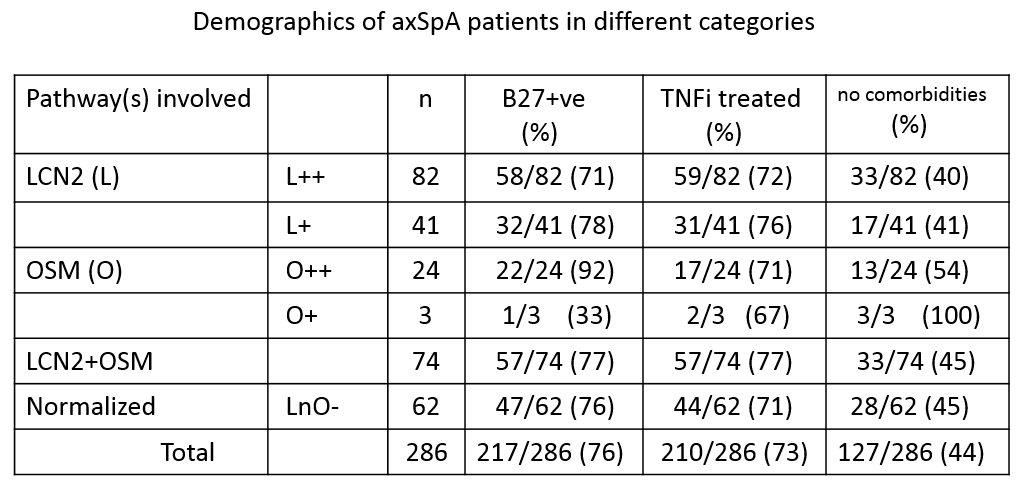


**Table S2**

**
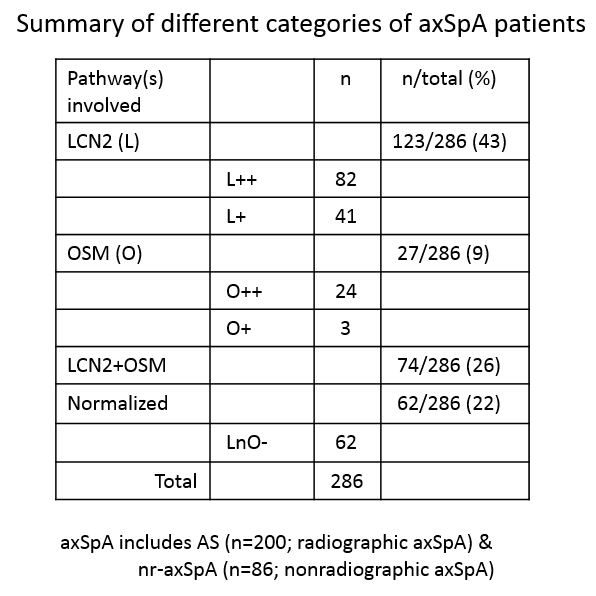
**

**Table S3**


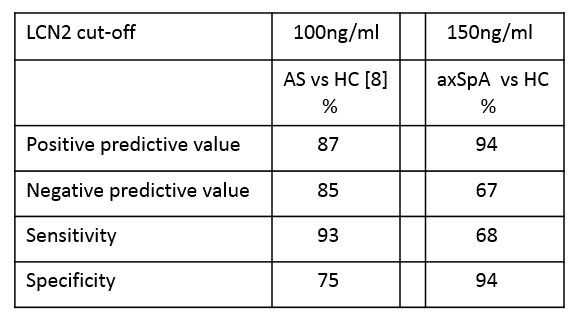


**Table S4**


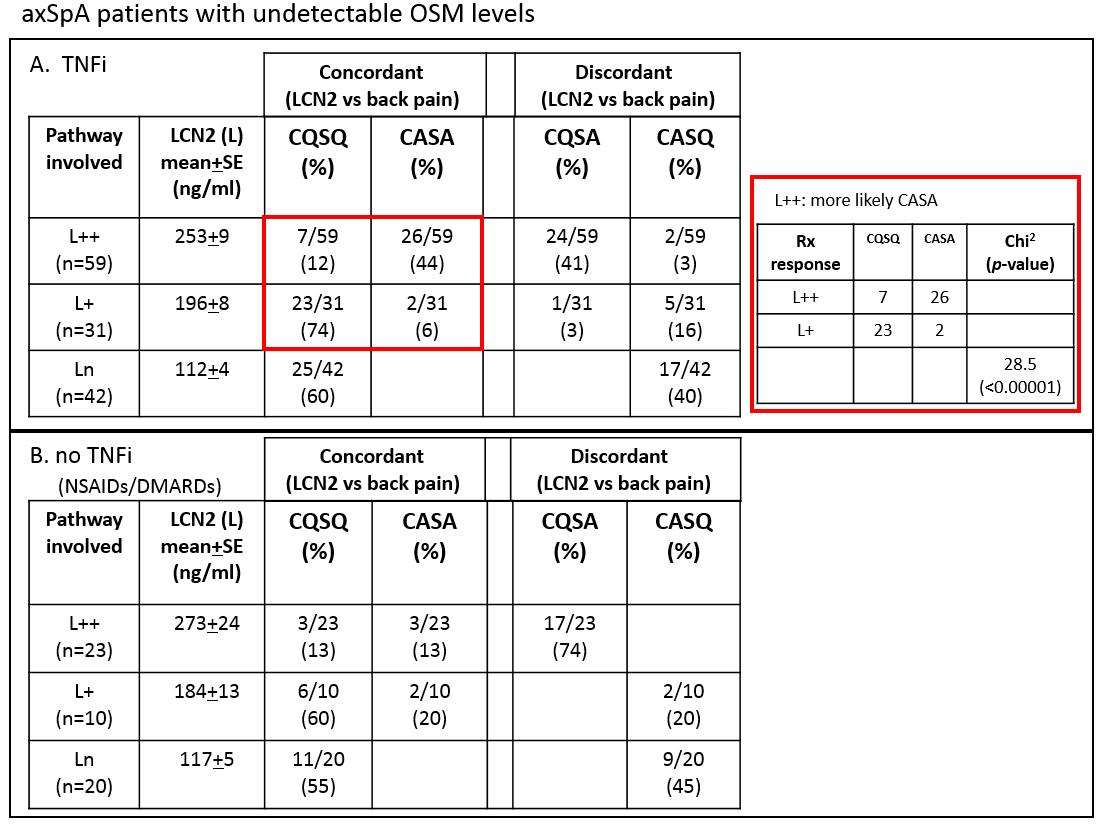


**Table S5**


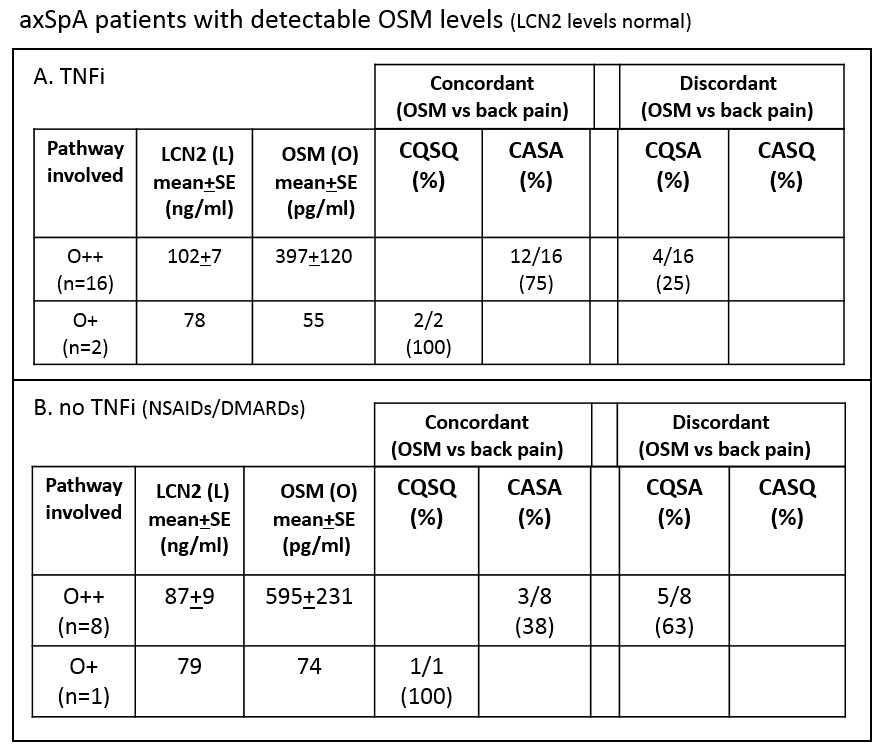


**Table S6**

**
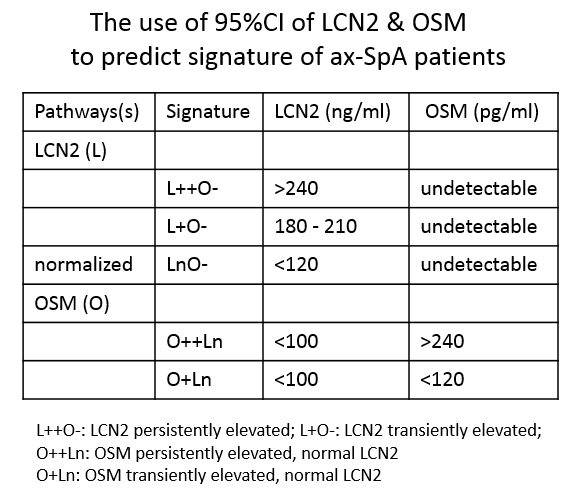
**

**Table S7**


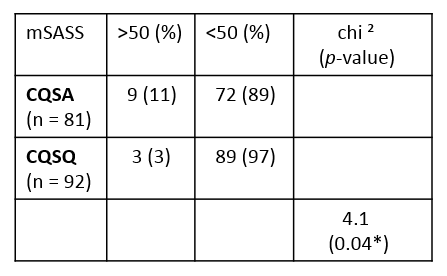

Supplement: Supplementary file 1 — Additional file 1: Figure S1. Correlation of CRP levels with MRI scores. Correlation of CRP levels with SPARCC SIJ scores and Berlin Spine scores in patients with the involvement of LCN2 pathway alone (L++) and with normal LCN2 (Ln). Pearson's correlation coefficient test was used. Table S1. Demographics of axSpA patients in different categories. Table S2. Summary of different categories of patients with the involvement of different pathway(s). Table S3. Sensitivity, specificity, positive and negative predictive values using different LCN2 cutoffs. Table S4. Treatment outcome in patients with LCN2 pathway alone (TNFi vs no TNFi). A. Comparison of OSM negative patients treated with TNFi: patients with persistent LCN2 elevation (L++) vs. transient LCN2 elevation (L+) vs. normal LCN2 (Ln). B. Comparison of OSM negative patients never received TNFi treatment: L++ vs. L+ vs. Ln patients. One-way analysis of variance followed by Bonferroni’s multiple comparison test and Pearson’s chi square test were used. Table S5. Treatment outcome in patients with OSM pathway alone (TNFi vs no TNFi). A. Comparison of patients with normal LCN2 treated with TNFi: patients with persistent OSM elevation (O++) vs transient OSM elevation (O+). LCN2 and OSM levels were compared between these two patient groups. B. Comparison of patients with normal LCN2 and never received TNFi treatment: O++ vs O+ patients. LCN2 and OSM levels were compared between these two patient groups. Table S6. The use of 95%CI of LCN2 and OSM to predict signature of axSpA patients. Table S7. Prevalence of patients having mSASS>50 in CQSA vs CQSQ subgroups. [file 13075_2021_2521_MOESM1_ESM.docx]
